# The adaptability of *Ulmus pumila* and the sensitivity of *Populus sibirica* to semi-arid steppe is reflected in the stem and root vascular cambium and anatomical wood traits

**DOI:** 10.3389/fpls.2024.1393245

**Published:** 2024-06-12

**Authors:** Anastazija Dimitrova, Angela Balzano, Enkhchimeg Tsedensodnom, Ser-Oddamba Byambadorj, Batkhuu Nyam-Osor, Gabriella Stefania Scippa, Maks Merela, Donato Chiatante, Antonio Montagnoli

**Affiliations:** ^1^ Department of Bioscience and Territory, University of Molise, Pesche, Italy; ^2^ Department of Seed Science and Forest Stands, Hans Em Faculty of Forest Sciences, Landscape Architecture and Environmental Engineering, Ss. Cyril and Methodius University in Skopje, Skopje, North Macedonia; ^3^ Department of Wood Science and Technology, Biotechnical Faculty, University of Ljubljana, Ljubljana, Slovenia; ^4^ Laboratory of Forest Genetics and Ecophysiology, School of Engineering and Applied Sciences, National University of Mongolia, Ulaanbaatar, Mongolia; ^5^ Laboratory of Silviculture, College of Agriculture and Life Science, Chungnam National University, Daejeon, Republic of Korea; ^6^ Laboratory of Environmental and Applied Botany, Department of Biotechnology and Life Science, University of Insubria, Varese, Italy

**Keywords:** Siberian elm, Siberian poplar, microcores, afforestation, xylem, xylogenesis, Mongolia

## Abstract

Afforestation success is measured by the tree establishment and growth capacity which contribute to a range of ecosystem services. In the Mongolian steppe, *Populus sibirica* and *Ulmus pumila* have been tested as candidate species for large afforestation programs, by analyzing their response to a combination of irrigation and fertilization treatments. While in temperate and Mediterranean forest ecosystems, xylogenetic studies provide insight into the trees’ plasticity and adaptability, this type of knowledge is non-existent in semi-arid regions, whose climatic features are expected to become a global issue. Furthermore, in general, a comparison between the stem and root response is scarce or absent. In the present study, we show that the anatomical traits of the vascular cambium and the xylem, from stem and root microcores, reflect the previously noted dependence of *P. sibirica* from irrigation – as they proportionally increase and the higher adaptability of *U. pumila* to drought – due to the reduced impact across all five characteristics. As the first wood anatomy study of these species in semiarid areas, future research is urgently needed, as it could be a tool for quicker understanding of species’ suitability under expected to be exacerbated semi-arid conditions.

## 1 Introduction

In the face of adverse climatic changes and projections for aridity increase - higher temperatures and reduced precipitation - the need for strategic management of arid and semi-arid areas is apparent (Singh and Chudasama, 2021), in many countries, including Mongolia. These challenges are further augmented by the nomadic pastoral systems, which dominantly utilize the space and scarce resources by overgrazing (Galvin et al., 2008; Liu et al., 2013; Hilker et al., 2014) and by the continuous northward shifts of ecological zone boundaries, overall resulting in increased land area of the desert steppe (Angerer et al., 2008). Afforestation is widely recognized as a counteractive measure that provides numerous ecosystem services, i.e., carbon sequestration, soil and water conservation, land reclamation and biodiversity support (Reisman-Berman et al., 2019; Liu et al., 2022a). The need for afforestation has been recognized as vital in Mongolia, thus encouraging the establishment of the *Green Belt* Plantation Project, a joint effort from the Mongolian and South Korean governments, aiming to deliver afforestation benefits in the critical arid and semi-arid areas of the country (Lee and Ahn, 2016; Nyam-Osor et al., 2021; Byambadorj et al., 2021a; Byambadorj et al., 2021b; Byambadorj et al., 2021c; Montagnoli et al., 2022; Avirmed et al., 2023). Yet, despite the potential of afforestation to mitigate some of the climatic extremes in semi-arid areas, there has been surprisingly little research on the various factors that contribute to the successful establishment of tree plantations in these regions (Yosef et al., 2018).

Species selection has a crucial role in afforestation as the plants’ eco-morphological and physiological traits determine their capacity for adaptation and plasticity, i.e., the potential for successful establishment and provision of ecosystem services (Dov et al., 2001; Díaz et al., 2016). In the drylands, experimental evaluation of woody species could significantly increase the afforestation benefits and facilitate region-specific decision-making, allowing for more stable and productive ecosystems (Reisman-Berman et al., 2019). To an extent, the challenging conditions in the drylands could also be mitigated by various management techniques, i.e., soil preparation and increased water and nutrient availability (Querejeta et al., 2001). Indeed, practices as preparatory fertilization can provide residual long-term effects on tree growth (From et al., 2015; Monteoliva et al., 2015; Smolander et al., 2022). As part of the *Green Belt* Plantation Project, a complex experimental design has been implemented to understand the impact of management (fertilization and irrigation) on the development of two species of interest, *Populus sibirica* Hort, Ex. Taush (Siberian poplar) and *Ulmus pumila* L. (Siberian elm). Ten years after the out-planting, several research methodologies have been used to evaluate the suitability of using these species for semi-arid afforestation and how they have been impacted by different combinations of fertilization and irrigation. *P. sibirica* was found to have a higher capacity for biomass production, thus sequestrating more carbon especially in the above-ground organs, and more rapid ground cover establishment (Byambadorj et al., 2021b). However, it was also found that the poplar is more sensitive to water shortage, a major constraint of semi-arid conditions (Byambadorj et al., 2021a). *U. pumila* was found to be more adapted to drought conditions, growing successfully without irrigation, i.e., with rainfall as the only water source (Byambadorj et al., 2021a; Byambadorj et al., 2021b). Furthermore, the elm was also negatively affected by fertilization treatments, expressed as reduced root biomass and plastic adaptation to prevailing wind (Nyam-Osor et al., 2021; Montagnoli et al., 2022). However, the current body of knowledge does not provide information on the impact of the environmental conditions and management practices on the cambial tissue and wood anatomical traits. The plant secondary growth is orchestrated by the cambium, longitudinal tissue present in both the above and belowground organs (Chiatante et al., 2021), whose complexity regarding its form and function has contributed to various definition attempts. In the present study, we rely on the definition proposed by the International Association of Wood Anatomists (IAWA, 1964) for the vascular cambium (VC) as *‘the actively dividing layer of cells that lies between, and gives rise to, secondary xylem and phloem’.* The VC is not static and the extent of its function depends on the position in the tree, the tree age, the climatic attributes of the habitat and the season, as well as potential internal signals and external stimuli, e.g., wounding, flooding, etc (Larson, 1994). Considering that the main activity of the VC is to divide and that these divisions are impacted by numerous factors, the products of the VC can be used to evaluate the tree’s adaptive potential. Gathering the information recorded in the wood structure allows us to project the dynamics of the cell development and link it with registered environmental conditions by analyzing the aforementioned products of the VC, the secondary xylem (the wood), and the secondary phloem (Čufar et al., 2011). In angiosperms, the differentiation of the cell functions in the wood has been considered as one of the tools for adaptation to many different habitats and environments, since xylem vessel elements have the sole function of continuously conducting water upwards; while the mechanical support is provided mainly by imperforated tracheary elements, i.e., libriform fibers (Rosell et al., 2017). Thus, xylogenesis studies have been used to understand the site and species-specific plasticity, as an indicator of the climatic impact (Prislan et al., 2018). In temperate regions, the regular seasonality eases the research focus on the cambial activity and tree-ring growth rates (Battipaglia et al., 2023). The main focus in this region has been on beech (*Fagus sylvatica* L.) due to its economic viability (e.g., Prislan et al., 2013; Prislan et al, 2018; Prislan et al, 2019; Arnič et al., 2021; Miranda et al., 2022). More recently, it has shifted to the semi-arid Mediterranean region, where the mild rainy winters and hot dry summers dictate a non-uniform tree growth, i.e., alternations of growth and dormancy during the year, which reflect as intra-annual density fluctuations in the tree rings (IADFs) (De Luis et al., 2007; De Micco et al., 2016; Balzano et al., 2018; Montagnoli et al., 2019; Battipaglia et al., 2023).

As the research regarding the wood traits of *P. sibirica* and *U. pumila* species of interest in Mongolia is limited, in a recent publication, our group first aimed to describe their anatomical traits through samples from the semi-arid steppe (Dimitrova et al., 2023). Briefly, *P. sibirica* exhibits the typical anatomy of poplar with diffuse porous wood, distinct growth ring boundaries, generally thin-walled fibers, and exclusively uniseriate rays (Wheeler, 2011; Wheeler et al., 2020; Dimitrova et al., 2023; InsideWood, 2023). Comparing the wood from the stem and the root, the root has slightly larger vessels and less distinct growth ring boundaries, possibly due to the IADFs (Wheeler, 2011; Wheeler et al., 2020; Dimitrova et al., 2023; InsideWood, 2023). *U. pumila* exhibits the typical anatomy of elm with ring porous wood, distinct growth ring boundaries, latewood vessels in the tangential bands, the common presence of vessel cluster and tylosis, vascular/vasicentric tracheids, thin- and thick-walled fibers and larger rays (4–10-seriate) of two distinct sizes (Wheeler, 2011; Wheeler et al., 2020; Dimitrova et al., 2023; InsideWood, 2023). Comparing the wood from the stem and the root, the root has less distinct growth ring boundaries and more earlywood vessels than the stem, with probable IADFs (Dimitrova et al., 2023).

Since in the Mongolian context, the semi-arid climate in the steppe is characterized by dry cold winters and hot summers during which 80–90% of the annual precipitation occurs (Byambadorj et al., 2021d), xylogenesis is limited to the period between early May and late August. As wood formation, i.e., radial growth, via the VC activity is a turgor-driven process (Ren et al., 2015; Hag Husein et al., 2021; Rahman et al., 2022), reduced water availability in semi-arid soils is the primary limiting factor for the plant growth. These observations, combined with previous results of the impact of irrigation on the two species in the Mongolian steppe, indicate that increased water availability might have a positive impact on the plant growth. Furthermore, some of the studies have also underlined the neutral and negative fertilization impact on the measured plant characteristics with a general reduction on shoot and root biomass development (Nyam-Osor et al., 2021; Byambadorj et al., 2021a; Byambadorj et al., 2021b; Byambadorj et al., 2021c; Montagnoli et al., 2022). While previous xylogenetic studies have underlined some of the wood formation traits impacted by the drought, these studies were focused solely on the stem of woody plants (e.g., Van Der Werf et al., 2007; Pasho et al., 2012; Giagli et al., 2016; D’Andrea et al., 2020) leaving knowledge gaps regarding the impact of fertilization on the belowground organs. Finally, the tap root growth is also orchestrated by the VC and impacted by the below-ground conditions which can significantly differ from the above-ground conditions in the semi-arid regions and impact vital root functions i.e., the ability to adapt to the environmental conditions, provide stability for the plant and water and nutrients uptake (Montagnoli et al., 2019; Karlova et al., 2021; Montagnoli et al., 2022).

In the present study, we hypothesize that for both species, although at a higher extent for *Poplar sibirica*, increasing the watering regimes will increase (*i*) the VC stack cell number and length (*ii*) the last wood increment and the vessel area of both early and late wood, and these increases (*iii*) will be of higher magnitude in the stem compared to the root. In the case of fertilization, we hypothesize that the above-mentioned VC and wood anatomical traits will be diminished for both species although to a higher extent for *Ulmus pumila*. Also, for both species, the fertilization effect is expected to be magnified by the increasing watering regimes.

To test this hypothesis, we measured the five indicated characteristics in microcore samples obtained from the stem and the root of both species of interest, which have been exposed to a total of 12 management practice combinations of fertilization (control – no fertilization, NPK, and compost) and irrigation (control – no irrigation, 2, 4 and 8 L h^-1^). The microcores were taken in the autumn, when the cambium activity had terminated, allowing for samples where all five characteristics would be preserved. The main objective of the study was to understand how the management practices impact the above- and below-ground plant development, since in semi-arid regions such as the Mongolian steppe the above- and below-ground conditions could greatly vary. Also, since microcores methodology is not yet commonly used in extreme terrains such as semi-arid steppe, this study would provide insights into the methodological approach for studying performance evaluation in afforestation programs.

## 2 Materials and methods

### 2.1 Site characteristics

**Figure 1:**
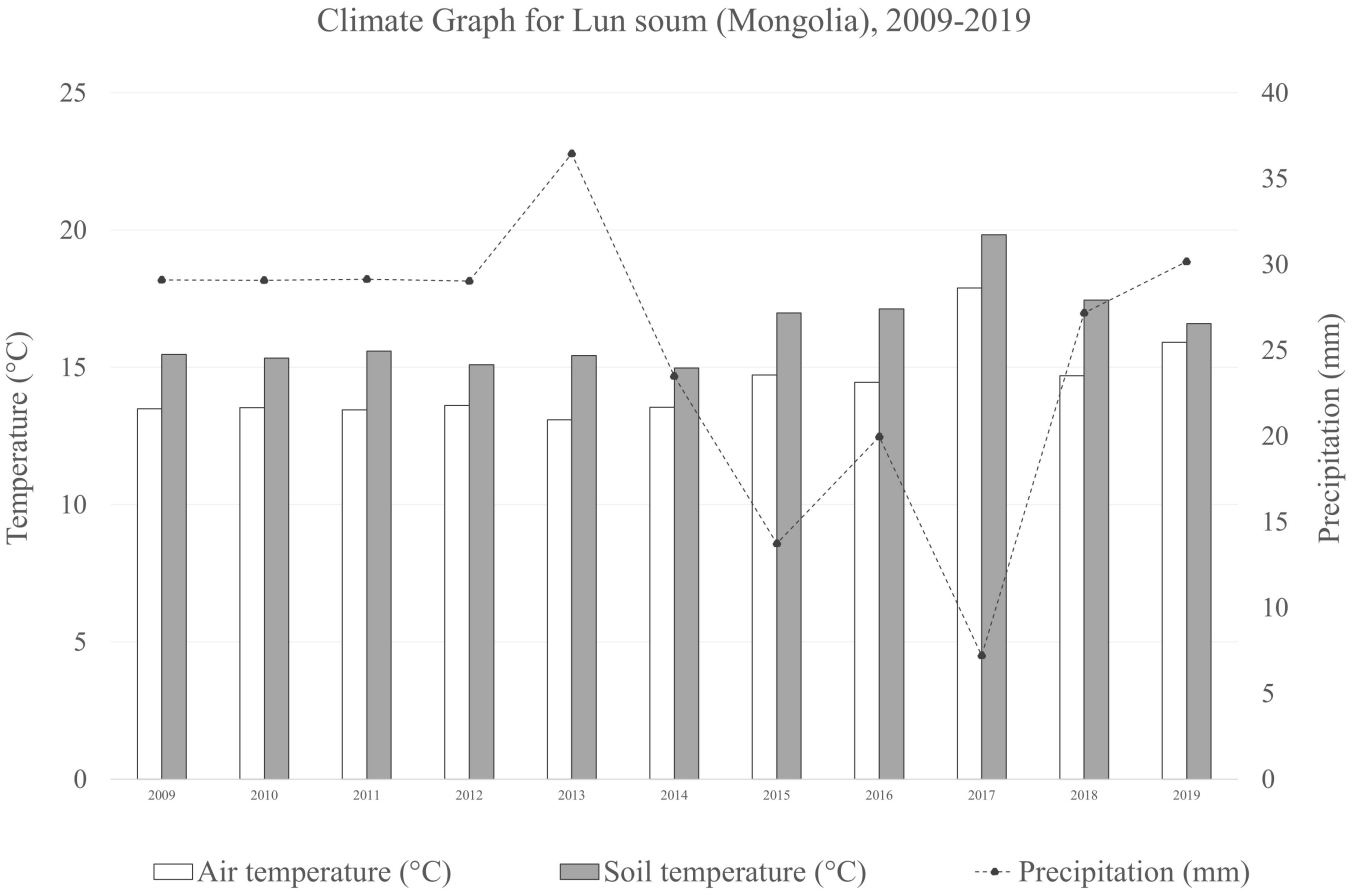
Climate graph the averaged air temperature, soil temperature and precipitation during the on-field growth period of the plants (2009–2019) of the sampling site in Lun soum (Mongolia).

The experimental site is located in Lun soum, Tuv province, Mongolia (47°52′15.43'′N, 105°10′46.4′'E), at an elevation of 1,130 m a.s.l. The site occupies 2 ha of the forest nursery of the South Korea-Mongolia joint *Green Belt* plantation project in the Middle Khalkha dry steppe region (Ulziykhutag, 1989), an area degraded due to intense livestock grazing. The area is mainly flat with Kastanozems (Loamic) soil type, immature, and lacking horizontal development with the top soil drier than the subsoil (Batkhishig, 2016).The vegetation is typical of the genuine dry bunchgrass steppe, dominated by xerophytic and meso-xerophytic graminoides (Lavrenko et al., 1991) and a semi-arid climate (**Figure 1**).

### 2.2 Plant material and experimental setup

**Figure 2:**
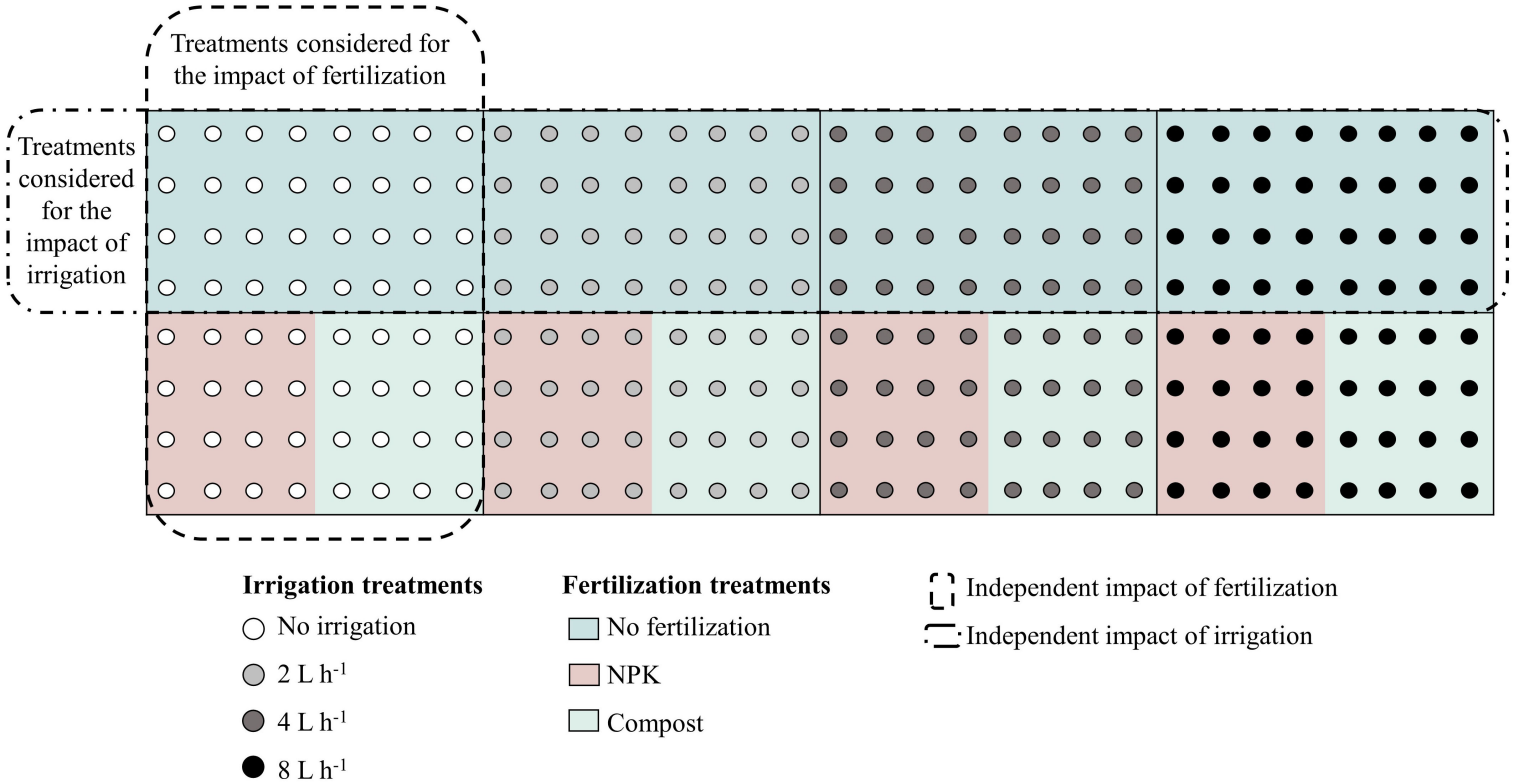
Schematic representation of irrigation and fertilization treatment combinations, indicated with the different circle colors and background colors, respectively. The outlined rectangles indicated the treatments that were considered for the analysis of the individual impact of the irrigation/fertilization. The same approach was applied for both species, *Populus sibirica* and *Ulmus pumila*. Figure adapted from Nyam-Osor et al. (2021).

Two-year-old saplings of *Ulmus pumila*, grown from seeds, and *Populus sibirica*, obtained from 20 cm cuttings, were acquired from the *Green Belt* Plantation Project nursery. After their initial cultivation in a greenhouse and acclimatization in an open nursery, in May 2011, they were transplanted into 60–70 cm-deep holes with a diameter of 50–60 cm. During the one-month acclimatization period, all saplings were uniformly irrigated with non-leakage (CNL) button drippers placed 10 cm from the stem of each tree. After the saplings acclimated, four different irrigation regimes were applied: no irrigation (control = 0 L h^-1^), 2 L h^-1 ^= 0.25 mm m^-2^, 4 L h^-1 ^= 0.5 mm m^-2^, and 8 L h^-1 ^= 1 mm m^-2^. Each watering session lasted for 5 hours and was conducted twice a week throughout the vegetative season (May to August). Additionally, two types of fertilizers were used as part of the management regimes – 500 g from either NPK or compost, were mixed with the natural soil before transplanting. NPK consisted of a solid granule mixture of nitrogen, phosphorus, and potassium, while the compost consisted of well-decomposed sheep manure. Twelve plots per plant species were prepared, including control and the various irrigation and fertilizer combinations. Each plot measured 20 × 10 m, with trees planted in north-south oriented rows north-south to maximize sunlight exposure and distance between rows and trees of 2.5 m (**Figure 2**).

### 2.3 Microcores processing, image acquisition, and measured anatomical parameters

In October 2019, the 10-year-old trees (plant height and stem diameter taken 5 cm above the ground line are reported in **Table 1**) were sampled with a Trephor tool, which provides for 2 mm diameter microcores (Rossi et al., 2006). Representative samples from each combination of fertilization and irrigation treatments (total of 12 combinations) were collected for both species. In the present study, the analysis was based on the four most preserved biological replicates, i.e., four microcores, from each treatment, resulting in a total of 96 microcores samples per species, 48 from the stem (5 cm above ground) and 48 from the root (5 cm below ground). All microcores were processed with an adapted protocol from Rossi et al. (2006); Prislan et al. (2013), and Balzano et al. (2019). In detail, the microcores were conserved initially in formaldehyde alcohol acetic acid solution (FAA) and later in 70% ethanol, at 4°C. Subsequently, they were embedded in paraffin, first by dehydration with ethanol and D-limonene (Bio Clear, Bio Optica, Milano, Italy), and then by immersion in liquid paraffin at 65°C (Paraplast plus, ORTH, Karlsruhe, Germany). Before embedding, the transverse orientation was marked on each microcore and used as an indicator for the positioning in the biocassettes. Once hardened, the paraffin blocks were trimmed to expose the wood, and after water immersion (at room temperature for 16–48 hours) microcore sections (9 µm thick) were cut with a Leica RM 2245 rotary microtome (Leica Microsystems, Wetzlar, Germany). Representative sections were placed on microscope slides that were previously treated with albumin, allowing for better adhesion. The slides were then dried at 75°C for 20 minutes and the residual paraffin was cleaned by gradual immersion in D-limonene and ethanol. Once cleaned, the sections were stained with a safranin (Merck, Darmstadt, Germany) (0.04%) and astra blue (Sigma-Aldrich, Steinheim, Germany) (0.15%) water mixture and permanently mounted on glass slides in Euparal (Bioquip Rancho Domingez, California). The sections were observed under a BX60 transmission light microscope (Olympus, Hamburg, Germany), their images were obtained with a digital camera (CAMEDIA, C4040, Olympus), and analyzed with the software AnalySIS 3.2. (Olympus) and Image J (Schneider et al., 2012).

**Table 1:** Plant height (H) and stem diameter (StD) of *Populus sibirica* and *Ulmus pumila* measured in 2019.

Treatment		*Populus sibirica*		*Ulmus pumila*	
Fertilization	Irrigation	H (cm)	StD (cm)	H (cm)	StD (cm)
No fertilization	No irrigation	197	6.8	183	3.8
	2 L h^-1^	336	6.7	193	4
	4 L h^-1^	342	7.1	213	4
	8 L h^-1^	428	7.9	232	5.2
NPK	No irrigation	288	5.4	140	3.6
	2 L h^-1^	272	6.3	222	5.2
	4 L h^-1^	279	6.8	200	5.2
	8 L h^-1^	431	8	207	4.6
Compost	No irrigation	255	4.8	198	4.2
	2 L h^-1^	334	6.3	240	4.9
	4 L h^-1^	339	7.1	188	4.1
	8 L h^-1^	372	7	201	4.8

**Figure 3:**
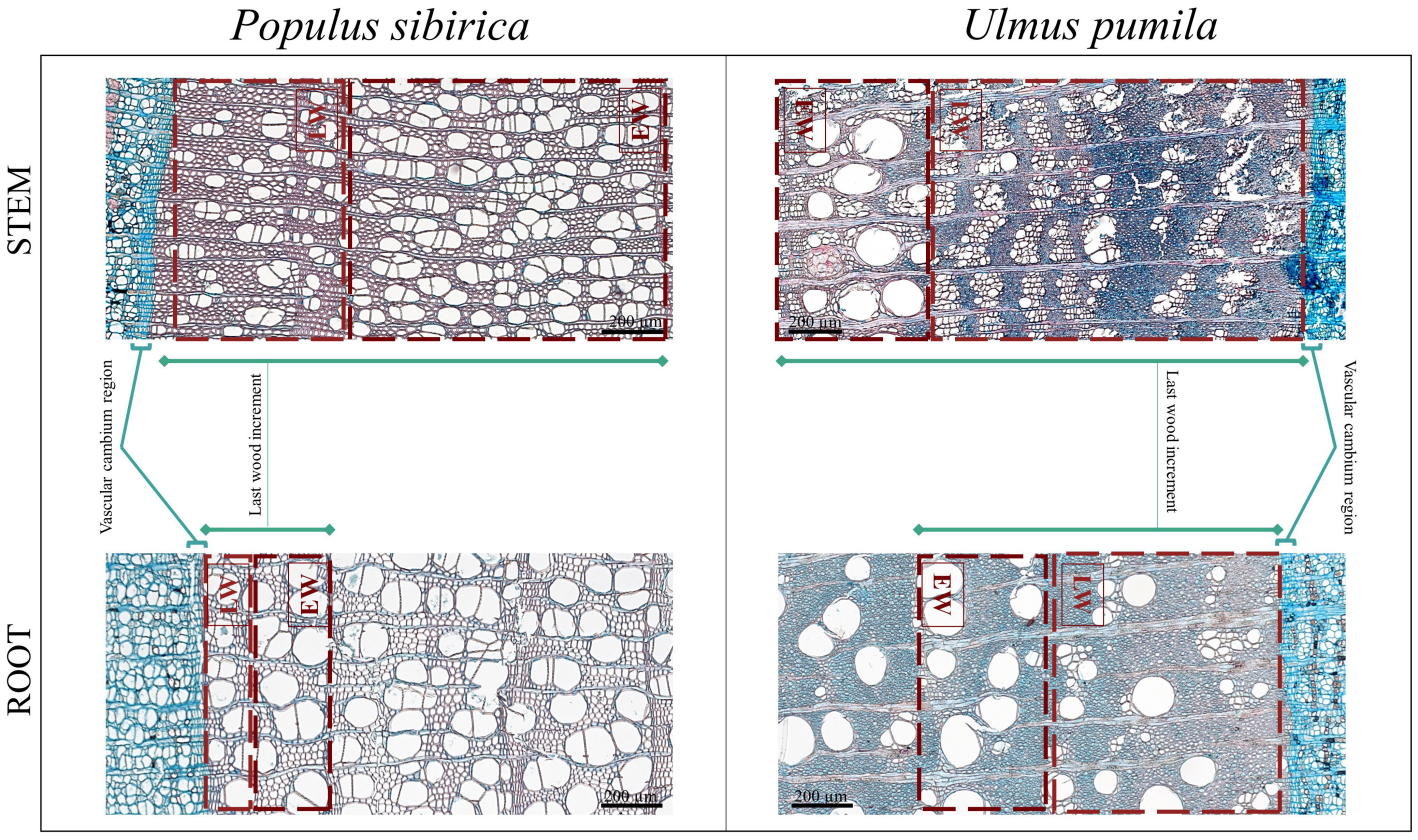
Microscopy images from *Populus sibirica* and *Ulmus pumila* stem and root microcores where the traits of interest have been marked. LW, Late Wood; EW, Early Wood.

The following anatomical traits were identified and counted/measured on the acquired microcore images (**Figure 3**):

*(i)* N-count (CCn) as the number of cambial cells in a stack in the cambial layer,
*(ii)* the length in µm (CCl) of the cambial cells stack,
*(iii)* the length in µm (LWI) of the last wood increment,
*(iv)* the area in µm^2^ of the xylem vessels in the early- (EWXA) and late-wood (LWXA) region.

Three technical replicates i.e., three separate measurements per sample, were taken in the case of CCn, CCl and LWI. The xylem vessels area was measured in the earlywood and latewood region and considered as a sum of 20 randomly selected vessels in both regions.

### 2.4 Statistical analysis

The statistical analysis was performed on a data set consisting of four biological replicates from the stem and the root of both species, for each of the 12 treatments. For each biological replicate, three technical replicates, i.e., three measurements, were taken in the case of CCn, CCl and LWI. For EWXA and LWXA, the final data represents a sum of 20 xylem vessels measured in the early and latewood section of the microcore, respectively (**Supplementary Material 1**). Linear mixed-effect model (LMM) was fitted for the CCn, CCl and LWI. The LMM considered the plant organ, fertilization and irrigation treatment as the fixed effect and accounted for the potential correlation within the replications using a random effect structure. Additionally, the generalized R-squared values were computed to assess the proportion of variance explained by the models. To perform pairwise comparisons and determine the differences between the levels of ‘dep.var’, the ‘difflsmeans’ function was employed (**Supplementary Material 2**). For EWXA and LWXA, the analysis was done using a linear regression model and a *post-hoc* Tukey’s HSD test (**Supplementary Material 2**). Both models, LMM and the linear regression model were run separately for each of the plant species, *Ulmus* and *Populus*, and applied to a 95% significance levels. All analysis was run in R (R Core Team, 2021).

## 3 Results

### 3.1 The independent impact of fertilization

#### 3.1.1 Vascular cambium traits

##### 3.1.1.1 Number of cambial cells

**Figure 4:**
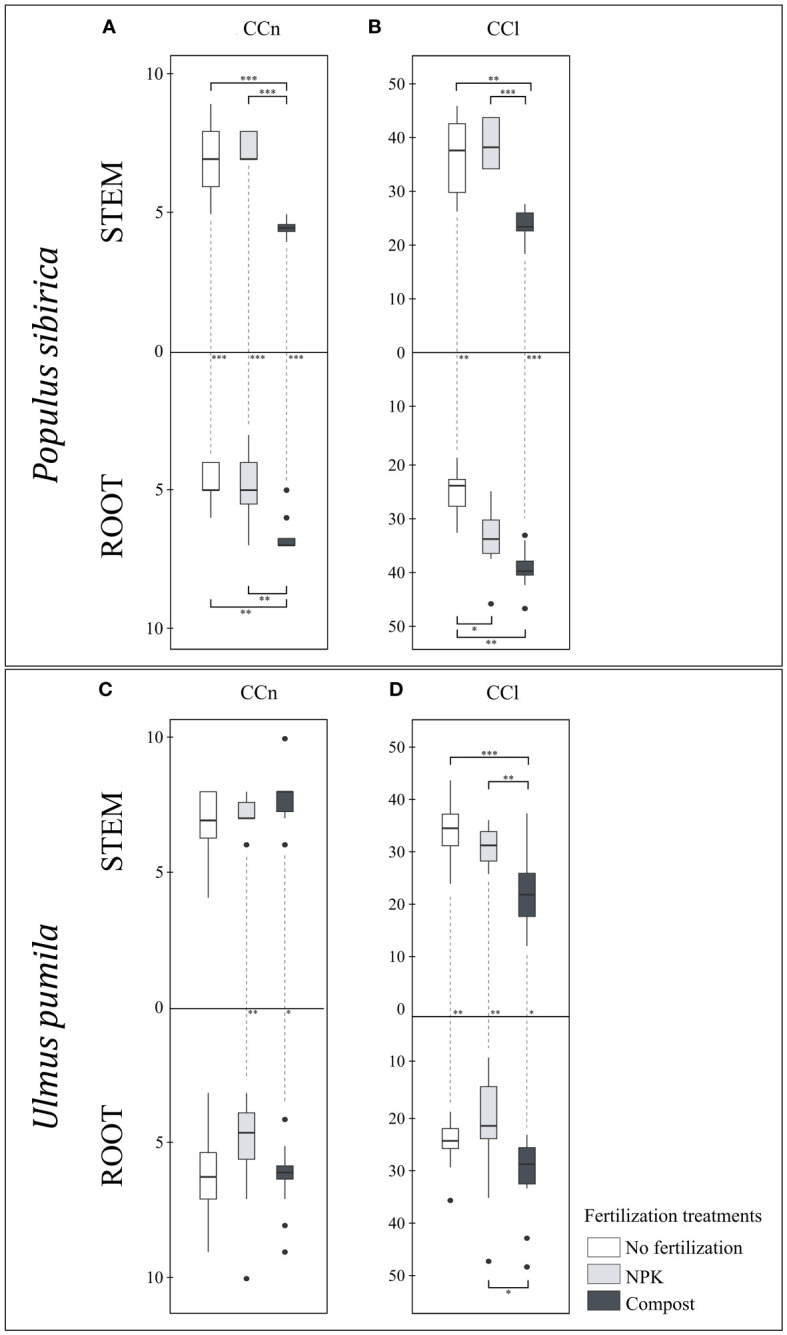
**(A)** The individual impact of fertilization on the stem and root number of cambial cells (N-count) - CCn in *Populus sibirica*. **(B)** The individual impact of fertilization on the stem and root length of the cambial cells stack (µm) – CCl in *Populus sibirica*. **(C)** The individual impact of fertilization on the stem and root number of cambial cells (N-count) - CCn in *Ulmus pumila*. **(D)** The individual impact of fertilization on the stem and root length of the cambial cells stack (µm) – CCl in *Ulmus pumila*. The vertical boxes present approximately 50% of the observations and the lines extending from each box are the upper and lower 25% of the distribution. Within each box, the solid horizontal line indicates the median. The dots represent the single data point. The significant differences based on the statistical analysis are indicated with symbols (p > 0.001 ‘***’, p < 0.01 ‘**’, p < 0.05 ‘*’). The whiskers above/below the boxes indicated the significant differences between the different fertilization treatments in the stem or root, respectively. The vertical dotted lines indicated the significant difference between the above- and below-ground response, treated with the same fertilization treatment.

In *P. sibirica*, the CCn was significantly affected by the fertilization treatments as the compost-treated trees had lower stem CCn but higher root CCn than the control trees and NPK-treated trees (**Figure 4A**). Regardless of the fertilization treatment, a significant difference was noted in the CCn between the stem and root. Both control and NPK-treated trees had higher stem CCn than the root, and, contrary to that, compost-treated trees had higher root CCn than the stem (**Figure 4A**). In *U. pumila*, the fertilization treatments had no significant impact on either the stem or the root CCn (**Figure 4C**). However, NPK- and compost-treated trees had higher stem CCn than the root (**Figure 4C**)

##### 3.1.1.2 Length of cambial cells stack

In *P. sibirica*, the stem CCl did not differ between the control and NPK-treated trees, while the compost-treated trees had the lowest CCl of all fertilization treatments (**Figure 4B**). The root CCl was highest and lowest, in the compost-treated and control trees, respectively, while the NPK-treated trees exhibited intermediate values (**Figure 4B**). Only with NPK fertilization, no significant difference for the CCl was observed between the stem and the root (**Figure 4B**). In the control, the CCl was higher in the stem than in the root, while in the compost, the CCl was higher in the root than in the stem (**Figure 4B**). In *U. pumila*, the compost-treated trees had significantly lower stem CCl compared to both control and NPK-treated trees (**Figure 4D**). However, the NPK-treated trees resulted in the lowest root CCl (**Figure 4D**). In the control and NPK-treated trees, the stem CCl was higher than the root, while in the compost-treated trees the stem CCl was lower than the root (**Figure 4D**).

#### 3.1.2 Anatomical wood traits

##### 3.1.2.1 Length of the last wood increment

**Figure 5:**
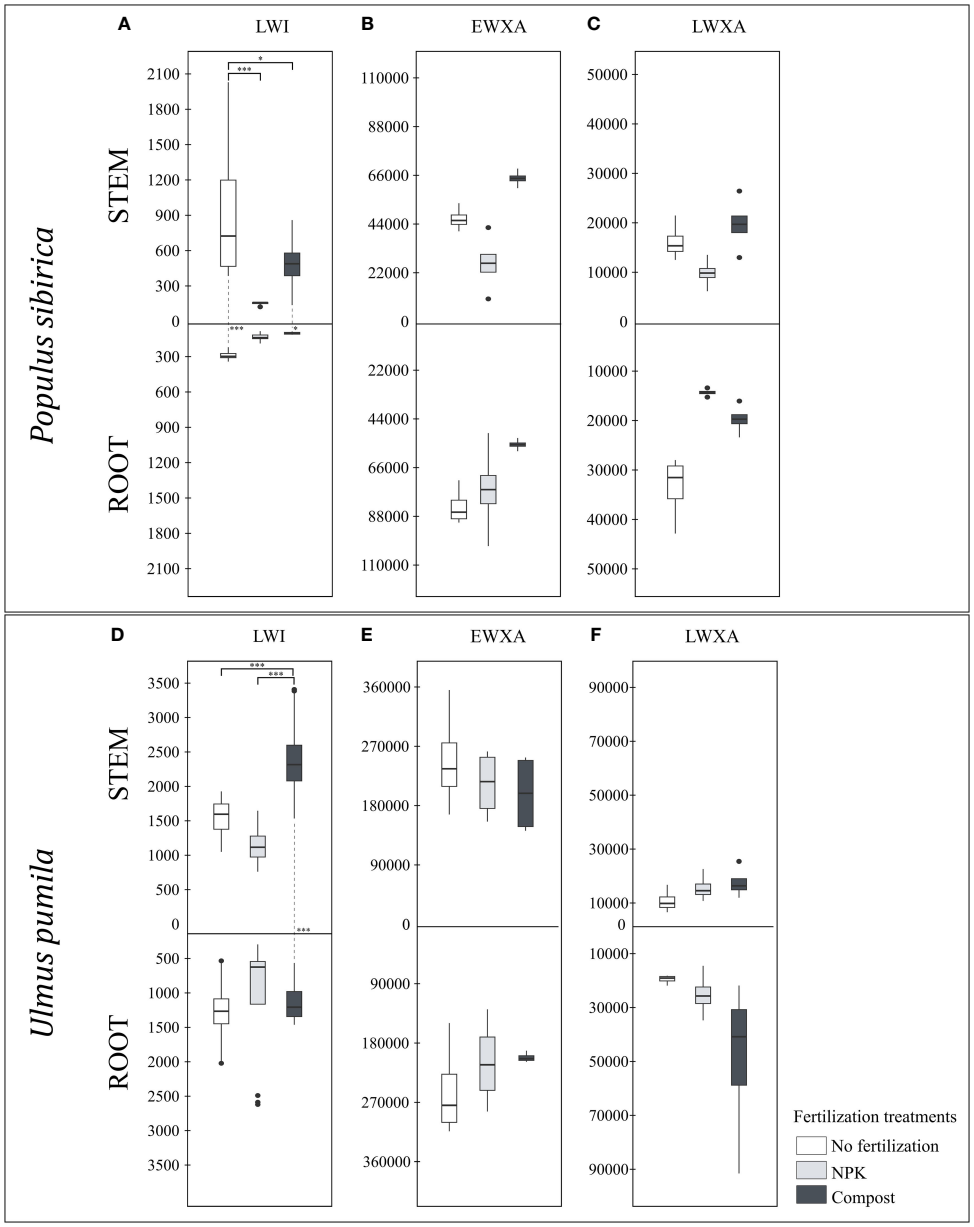
**(A)** The individual impact of fertilization on the stem and root length of the last wood increment (µm) – LWI in *Populus sibirica*. **(B)** The individual impact of fertilization on the stem and root early wood xylem area (µm^2^) - EWXA in *Populus sibirica*. **(C)** The individual impact of fertilization on the stem and root late wood xylem area (µm^2^) – LWXA in *Populus sibirica*. **(D)** The individual impact of fertilization on the stem and root length of the last wood increment (µm) – LWI in *Ulmus pumila*. **(E)** The individual impact of fertilization on the stem and root early wood xylem area (µm^2^) - EWXA in *Ulmus pumila*. **(F)** The individual impact of fertilization on the stem and root late wood xylem area (µm^2^) – LWXA in *Ulmus pumila*. The vertical boxes present approximately 50% of the observations and the lines extending from each box are the upper and lower 25% of the distribution. Within each box, the solid horizontal line indicates the median. The dots represent the single data point. The significant differences based on the statistical analysis are indicated with symbols (p > 0.001 ‘***’, p < 0.05 ‘*’). The whiskers above/below the boxes indicated the significant differences between the different fertilization treatments in the stem or root, respectively. The vertical dotted lines indicated the significant difference between the above- and below-ground response, treated with the same fertilization treatment.

In *P. sibirica*, the stem LWI in the control was significantly higher than both NPK- and compost-treated trees (**Figure 5A**). Regardless of the fertilization treatments, the root LWI was not impacted (**Figure 5A**). Only in the NPK-treated trees the stem LWI was not significantly different from the root (**Figure 5A**). However, for both the control and the compost-treated trees, the stem LWI was significantly higher than the root (**Figure 5A**). In *U. pumila*, a significant difference due to the fertilization was observed only in the stem LWI, highest in compost-treated trees (**Figure 5D**). Furthermore, only the compost-treated trees had higher stem LWI than the root, while in the control and NPK-treated trees, no significant differences were noted (**Figure 5D**).

##### 3.1.2.2 Early- and latewood xylem area

In both *P. sibirica* and *U. pumila*, neither in the stem nor in the root, and between these two organs, were the EWXA and LWXA were affected by the fertilization treatment (**Figures 5B, C, E, F**).

### 3.2 The independent impact of irrigation and the interplay between irrigation and fertilization

#### 3.2.1 Vascular cambium traits

##### 3.2.1.1 Number of cambial cells

**Figure 6:**
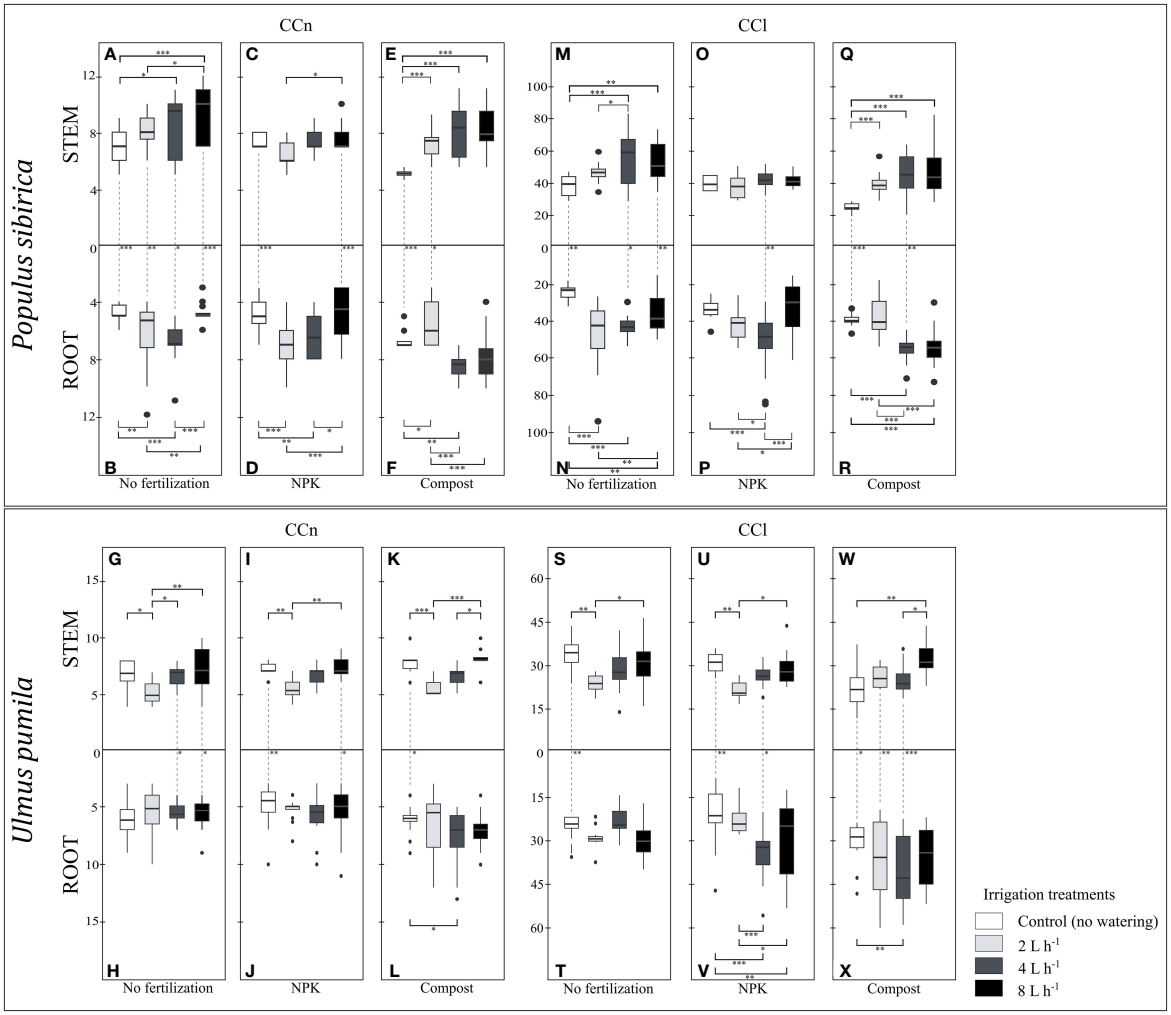
**(A–F)** The independent impact of irrigation and the interplay between irrigation and fertilization on the stem and root number of cambial cells (N-count) - CCn in *Populus sibirica*. **(G–L)** The independent impact of irrigation and the interplay between irrigation and fertilization on the stem and root number of cambial cells (N-count) - CCn in *Ulmus pumila*. **(M–R)** The independent impact of irrigation and the interplay between irrigation and fertilization on the stem and length of the cambial cells stack (µm) – CCl in *Populus sibirica*. **(S–X)** The independent impact of irrigation and the interplay between irrigation and fertilization on the stem and root length of the cambial cells stack (µm) – CCl in *Ulmus pumila*. The vertical boxes present approximately 50% of the observations and the lines extending from each box are the upper and lower 25% of the distribution. Within each box, the solid horizontal line indicates the median. The dots represent the single data point. The significant differences based on the statistical analysis are indicated with symbols (p > 0.001 ‘***’, p < 0.01 ‘**’, p < 0.05 ‘*’). The whiskers indicated the significant differences between the different fertilization treatments in the stem or root, respectively. The vertical dotted lines indicated the significant difference between the above- and below-ground response, treated with the same fertilization treatment.

**Figure 7:**
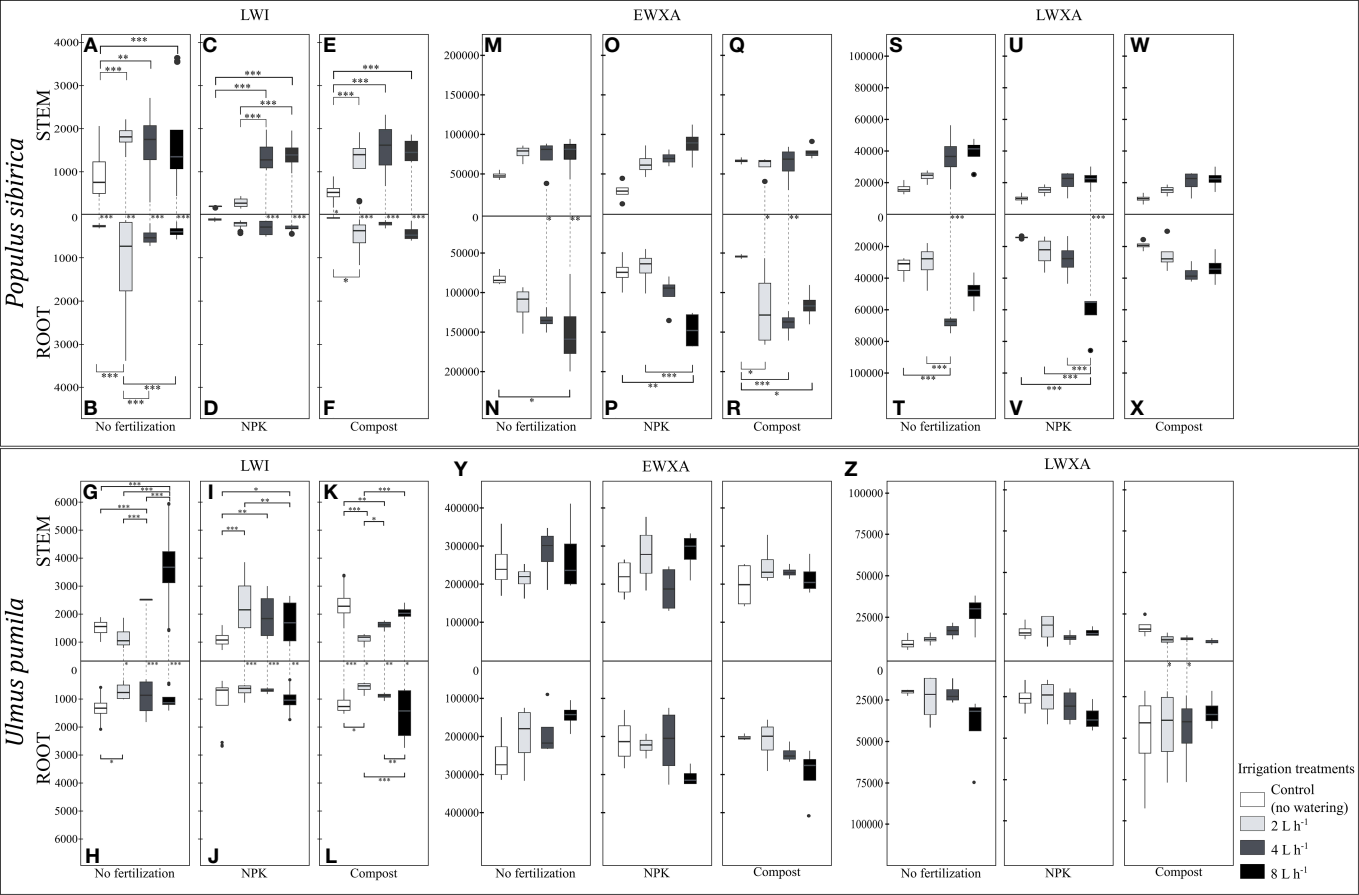
**(A–F)**. The independent impact of irrigation and the interplay between irrigation and fertilization on the stem and root length of the last wood increment (µm) – LWI in *Populus sibirica*. **(G–L)** The independent impact of irrigation and the interplay between irrigation and fertilization on the stem and root length of the last wood increment (µm) – LWI in *Ulmus pumila*. **(M–R)** The independent impact of irrigation and the interplay between irrigation and fertilization on the stem root early wood xylem area (µm^2^) - EWXA in *Populus sibirica*. **(S–X)** The independent impact of irrigation and the interplay between irrigation and fertilization on the stem and root late wood xylem area (µm^2^) – LWXA in *Populus sibirica*. **(Y)** The independent impact of irrigation and the interplay between irrigation and fertilization on the stem root early wood xylem area (µm^2^) - EWXA in *Ulmus pumila*. **(Z)** The independent impact of irrigation and the interplay between irrigation and fertilization on the stem and root late wood xylem area (µm^2^) – LWXA in *Ulmus pumila*. The vertical boxes present approximately 50% of the observations and the lines extending from each box are the upper and lower 25% of the distribution. Within each box, the solid horizontal line indicates the median. The dots represent the single data point. The significant differences based on the statistical analysis are indicated with symbols (p > 0.001 ‘***’, p < 0.01 ‘**’, p < 0.05 ‘*’). The whiskers indicated the significant differences between the different fertilization treatments in the stem or root, respectively. The vertical dotted lines indicated the significant difference between the above- and below-ground response, treated with the same fertilization treatment.

When no fertilization was applied, *P. sibirica* trees irrigated with 2 L h^-1^ regime did not exhibit different stem CCn values from both the control-irrigation and 4 L h^-1^ (**Figure 6A**), while the latter one had significantly higher stem CCn from the control-irrigation (**Figure 6A**). With the 8 L h^-1^ irrigation regime, the stem CCn was similar to the 4 L h^-1^ irrigation regime, and both were significantly higher than the stem CCn of the control-irrigation (**Figure 6A**). Both the 2 and 4 L h^-1^ irrigation regimes resulted in similar root CCn values, which were significantly higher than the control-irrigation (**Figure 6B**). With the 8 L h^-1^ irrigation regime, the root CCn was lower than both the 2 and 4 L h^-1^ irrigation regimes, but similar to the control values (**Figure 6B**). The control-irrigation resulted in significantly higher stem CCn than the root and these differences were maintained across all different irrigation regimes (**Figures 6A, B**). In NPK-treated *P. sibirica* trees, the stem CCn were similar between all four irrigation regimes; only between the 2 and 8 L h^-1^ irrigated trees, a higher stem CCn in the latter was observed (**Figure 6C**). With the NPK-fertilization, the root CCn of the control-irrigation and 8 L h^-1^ irrigation regime were similar to each other, however, both were significantly lower from the root CCn of the 2 and 4 L h^-1^ irrigation regimes (**Figure 6D**). Furthermore, both the control-irrigation and 8 L h^-1^ irrigation regime had significantly higher stem CCn than the root (**Figures 6C, D**). In compost-treated *P. sibirica* trees, all three watering regimes had similar stem CCn values, which were significantly higher than the control-irrigation (**Figure 6E**). The 2 L h^-1^ irrigation regime resulted in lower root CCn than the control and the other two irrigation regimes (**Figure 6F**). Furthermore, the control-irrigation had lower root CCn the 4 L h^-1^ irrigation regime, which in turn has similar root CCn to the 8 L h^-1^ irrigation regime (**Figure 6F**). When less water was available (i.e., control-irrigation and 2 L h^-1^ irrigation regime), a significant difference was observed between the stem and the root: in the control-irrigation, the stem CCn was lower than the root, and with the 2 L h^-1^ irrigation regime, the stem CCn was higher than the root (**Figures 6E, F**). In *U. pumila*, when no fertilization was applied, the 2 L h^-1^ irrigation had significantly lower stem CCn, compared to the control-irrigation and the two higher irrigation regimes (4 and 8 L h^-1^) (**Figure 6G**). When no fertilization treatment was applied, the irrigation regimes did not have a significant impact on the root CCn (**Figure 6H**). With the 4 L h^-1^ and 8 L h^-1^ irrigation regime, the stem CCn was significantly higher than the root; however, no differences between these two organs were observed in the control-irrigation and the 2 L h^-1^ irrigation regime (**Figures 6G, H**). In NPK-treated *U. pumila* trees, the stem CCn was lowest with the 2 L h^-1^ irrigation regime, which showed to be significantly lower than the control-irrigation and the 8 L h^-1^ irrigation regime, but not different from the 4 L h^-1^ irrigation regime (**Figure 6I**). The irrigation regimes combined with the NPK fertilization did not impact the root CCn (**Figure 6J**). With control-irrigation and when the highest watering regime was applied (8 L h^-1^), the NPK fertilization contributed to a significantly higher stem CCn than the root; while no differences were observed with the 2 and 4 L h^-1^ irrigation regimes (**Figures 7I, J**). In compost-treated *U. pumila* trees, the stem CCn did not differ between the control-irrigation and the 8 L h^-1^ irrigation (**Figure 6K**). However, the stem CCn with the 2 L h^-1^ irrigation regime was significantly lower than both the control-irrigation and 8 L h^-1^ irrigation regime; the latter was also significantly higher than the 4 L h^-1^ irrigation regime (**Figure 6K**). Only the root CCn in the control-irrigation was significantly lower than the 4 L h^-1^ irrigation regime (**Figure 6L**). Furthermore, only in the control-irrigation, the stem CCn was significantly higher than the root, and no significant differences were observed between the two organs with the other three irrigation regimes (**Figures 6K, L**).

##### 3.2.1.2 Length of cambial cells stack

In *P. sibirica*, when no fertilization was applied, the 4 L h^-1^ and 8 L h^-1^ irrigation regimes had similar stem CCl, significantly higher than both the control-irrigation and 2 L h^-1^ irrigation regime (**Figure 6M**). No differences were observed between the control-irrigation and 2 L h^-1^ irrigation regime (**Figure 6M**). All three irrigation regimes contributed to significantly higher root CCl than the control-irrigation (**Figure 6N**). Furthermore, the 8 L h^-1^ irrigation regime resulted in similar root CCl to the 4 L h^-1^ irrigation regime, yet both had lower root CCl than the 2 L h^-1^ irrigated trees (**Figure 6N**). The control-irrigation, 4 L h^-1^ and 8 L h^-1^ irrigation regimes had significantly higher stem CCl than the root, while no difference between these two organs was observed with the 2 L h^-1^ irrigation regime (**Figures 6M, N**). In NPK-treated *P. sibirica* trees, the irrigation regime did not contribute to any differences in the stem CCl (**Figure 6O**). The 4 L h^-1^ irrigation regime had higher root CCl than the control-irrigation, 2 L h^-1,^ and 8 L h^-1^ irrigation regimes (**Figure 6P**). Furthermore, while no significant differences were observed between the control-irrigation and 8 L h^-1^ irrigation regime root CCl, the latter was significantly lower than the 2 L h^-1^ irrigation regime root CCl (**Figure 6P**). Only with the 4 L h^-1^ irrigation regime there was a significant difference between the stem and root, with the root CCl being higher than the stem (**Figures 6O, P**). In compost-treated *P. sibirica* trees, the control-irrigation had lower stem CCl than all the irrigation regimes, which in turn had similar CCl between each other (**Figure 6Q**). The root CCl was similar between the control-irrigation and the 2 L h^-1^, and in both cases, it was lower than the root CCl of the 4 L h^-1^ and 8 L h^-1^ irrigation regimes (**Figure 6R**). In the control-irrigation and 4 L h^-1^ irrigation regimes, the stem CCl was lower than the root (**Figures 6Q, R**). In *U. pumila*, when no fertilization was applied, the stem CCl of the 2 L h^-1^ irrigation regime was significantly lower than the control-irrigation and the 8 L h^-1^ irrigation regime, while no significant differences were observed between the stem CCl of the control-irrigation, 4 L h^-1^, and 8 L h^-1^ irrigation regime (**Figure 6S**). The irrigation regime had no impact on the root CCl (**Figure 6T**). Furthermore, only in the control-irrigation, the stem CCl was significantly higher than the root (**Figures 6S, T**). In NPK-treated *U. pumila* trees, similarly to the stem when no fertilization treatment was applied, the stem CCl with the 2 L h^-1^ irrigation regime was significantly lower than the control-irrigation and the 8 L h^-1^irrigation regime, while no differences were observed between the stem CCl of the control-irrigation, 4 L h^-1^ and 8 L h^-1^ irrigation regime (**Figure 6U**). In the NPK-treated root, the control-irrigation had similar root CCl to the 2 L h^-1^ irrigation regime; however, both treatments had a significantly lower root CCl than the 4 L h^-1^ and 8 L h^-1^ irrigation regimes (**Figure 6V**). In the control-irrigation and the 4 L h^-1^ irrigation regime, a significant difference was observed between the stem and the root – higher stem CCl in the former, and lower stem CCl in the latter irrigation regime (**Figures 6U, V**). In compost-treated *U. pumila* trees, the stem CCl of the control-irrigation and the 4 L h^-1^ irrigation regime were significantly lower than the 8 L h^-1^ irrigation regime (**Figure 6W**). Only the control-irrigation root CCl was significantly lower than the 4 L h^-1^ irrigation (**Figure 6X**). Between the two organs, in the control-irrigation the stem CCl was higher than the root; however, for both the 2 and 4 L h^-1^ irrigation regimes, the stem CCl was lower than the root (**Figures 6W, X**).

#### 3.2.2 Anatomical wood traits

##### 3.2.2.1 Length of the last wood increment

When no fertilization treatment was applied, in *P. sibirica*, the stem LWI in the control-irrigation was significantly lower than the rest of the irrigation regimes, which were similar to each other (**Figure 7A**). The root LWI was higher with the 2 L h^-1^ irrigation regime than the rest of the irrigation regimes, which were also similar to each other (**Figure 7B**). Regardless of the irrigation regime, the stem LWI was significantly higher than the root (**Figures 7A, B**). In NPK-treated *P. sibirica* trees, the stem LWI was similar between the control-irrigation and the 2 L h^-1^ irrigation regime, and in both cases, it was lower than the stem LWI of the 4 L h^-1^ and 8 L h^-1^ irrigation regimes (**Figure 7C**). The root LWI was not impacted by the irrigation regime (**Figure 7D**). Only as the water availability increased, i.e., 4 L h^-1^ and 8 L h^-1^ irrigation regimes, the stem LWI was higher than the root (**Figures 7C, D**). In compost-treated *P. sibirica* trees, the stem LWI of the control-irrigation was lower than the three irrigation regimes, which in turn were similar to each other (**Figure 7E**). The root LWI of the 2 L h^-1^ irrigation regime was highest, but this showed significant only when compared to the control-irrigation (**Figure 7F**). Regardless of the irrigation regime, the stem LWI was significantly higher than the root (**Figures 7E, F**). In *U. pumila*, when no fertilization treatment was applied, the stem LWI of the 8 L h^-1^ irrigation regime was significantly higher than the control-irrigation and the other two irrigation regimes (**Figure 7G**). The LWI of the 4 L h^-1^ irrigation regime was significantly higher than both the control-irrigation and the 2 L h^-1^ irrigation regime (**Figure 7G**). In the root, the only significant difference was between the control-irrigation and the 2 L h^-1^ irrigation regime, with higher root LWI in the former (**Figure 7H**). Except in the control-irrigation, in all three irrigation regimes, the stem LWI was higher than the root (**Figures 7G, H**). In the NPK-treated *U. pumila* trees, the stem LWI in the control-irrigation was significantly lower than all three irrigation regimes, and the stem LWI of the 2 L h^-1^ irrigation regime was higher than with the 8 L h^-1^ irrigation regime (**Figure 7I**). No significant differences in the root LWI between the irrigation regimes were noted (**Figure 7J**). Except in the control-irrigation, the combination of the NPK fertilization and the irrigation regimes contributed to a higher stem LWI than the root (**Figures 7I, J**). In compost-treated *U. pumila*, the stem LWI in the control-irrigation was significantly higher than the 2 L h^-1^ and 4 L h^-1^ irrigation regimes (**Figure 7K**). Furthermore, the stem LWI of the 2 L h^-1^ irrigation regime was also significantly lower than the 4 L h^-1^ and 8 L h^-1^ irrigation regimes (**Figure 7K**). The root LWI of the control-irrigation was significantly higher than the 2 L h^-1^ irrigation regime while the 8 L h^-1^ irrigation regime was higher than both the 2 and 4 L h^-1^ irrigation regime root LWI (**Figure 7L**). Combined with the compost fertilization, in all four irrigation regimes, the stem LWI was significantly higher than the root (**Figures 7K, L**).

##### 3.2.2.2 Early- and latewood xylem area

In *P. sibirica*, neither the stem EWXA nor the stem LWXA was significantly impacted by any combination of the irrigation regimes and fertilization treatments (**Figures 7M, O, Q, S, U, W**). However, when no fertilization was applied, the root EWXA of the control-irrigation was significantly lower than the 8 L h^-1^ irrigation regime, which in turn was similar to the root EWXA of the 2 L h^-1^ and 4 L h^-1^ irrigation regimes (**Figure 7N**). Only with the 4 L h^-1^ and 8 L h^-1^ irrigation regimes, the root EWXA was significantly higher than the stem (**Figures 7M, N**). In NPK-treated *P. sibirica* trees, the root EWXA of the 8 L h^-1^ irrigation regime was significantly higher than both the control-irrigation and the 2 L h^-1^ irrigation regime (**Figure 7P**). No significant differences between the two organs were observed for any of the irrigation regimes when NPK fertilization was applied (**Figures 7O, P**). In compost-treated *P. sibirica* trees, the root EWXA of the control-irrigation was significantly lower than all the irrigation regimes (**Figure 7R**). In the case of 2 L h^-1^ and 4 L h^-1^ irrigation regimes, the root EWXA was significantly higher than the stem (**Figures 7Q, R**). When no fertilization was applied, the root LWXA of *P. sibirica* was significantly higher with the 4 L h^-1^ irrigation regime than both the control-irrigation and the 2 L h^-1^ irrigation regime (**Figure 7T**). Furthermore, only with the 4 L h^-1^ irrigation regime a significant difference was observed regarding the LWXA as the root LWXA was higher than the stem (**Figures 7S, T**). In NPK-treated *P. sibirica* trees, the root LWXA of the 8 L h^-1^ irrigation regime was significantly higher than the control-irrigation, the 2 and 4 L h^-1^ irrigation regimes, which were similar to each other (**Figure 7V**). Additionally, only in the case of the 8 L h^-1^ irrigation regime, the root LWXA was significantly higher than the stem (**Figures 7U, V**). In compost-treated *P. sibirica* trees, no significant difference regarding the LWXA due to the irrigation regimes was observed between the stem, between the root, and between these two organs (**Figures 7W, X**). In *U. pumila*, neither the EWXA nor the LWXA was significantly impacted by any combination of the irrigation regimes and fertilization treatments in all three comparisons, between the stems, between the roots, and between these two organs (**Figures 7Y, Z**).

## 4 Discussion

In semi-arid areas, drought tolerance and capacity for faster growth and establishment have been the main tree species selection criteria (Dov et al., 2001). This approach has also been used a decade ago, when selecting *P. sibirica* and *U. pumila*, for the *Green Belt* Plantation Project in the Mongolian semi-arid areas (Khaulenbek and Kang, 2017; Byambadorj et al., 2021b). The plants’ ability to adjust and survive environmental challenges is, in part, due to their phenotypic plasticity, reflected in the VC and xylem anatomy (Plavcová and Hacke, 2012), e.g., increased annual tree ring length and more cambial cell layers during dormancy are associated with healthy trees (Perez-de-Lis et al., 2017), while drought conditions cause decrease of the annual tree rings length (Rahman et al., 2022). These changes are not limited to the above-ground organs, although the woody stem has been the main point of interest. Indeed Liu et al. (2022b), concluded that, while climate warming does not have a significant impact on the start, end, and length of the growing season in above-ground plant organs, in below-ground organs it can delay the start of the growing season, indicating a higher sensitivity, and a disbalance between the above- and below-ground response. It has been previously shown that *P. sibirica* and *U. pumila* respond differently to the fertilization and irrigation treatments (Nyam-Osor et al., 2021; Byambadorj et al., 2021a; Byambadorj et al., 2021b). Thus, the present study employed a xylogenetic approach to analyze how these differences are reflected in the stem and root vascular cambium and anatomical wood traits.

### 4.1 Vascular cambium traits

Since fertilization has been shown to enhance plant growth, short-term and long-term (Landsberg and Sands, 2011; From et al., 2015; Monteoliva et al., 2015), we might assume that increased nutrient availability would also lead to an increased number of cambial cells, as well as their size. When *P. sibirica* trees were not watered, both the CCn and CCl in compost-treated trees were lowest in the stem and highest in the root, compared to both control (no fertilization) and NPK-treated trees. Furthermore, control and compost-treated trees showed different VC traits between the stem and the root, in the former the priority was the stem VC, while in the latter it was the root. This control response was similar to the NPK-treated *P. sibirica*, as the stem CCn and CCl are higher than the root. Therefore, looking at the individual impact of fertilization, our hypothesis can be partially confirmed regarding *P. sibirica* as only the compost treatment impacted the VC traits. Similarly, in *U. pumila*, the compost-treated trees also had the lowest stem CCl. While the root CCl of *U. pumila* was also highest in the compost-treated trees, this was significant only regarding the NPK-treated trees, and not the control. For the elm, the stem VC consistently benefited only from NPK fertilization and as the sole impact was on the CCl, we reject the hypothesis that the fertilization causes diminishing of the VC traits. It seems that in both species, the two different fertilization types have a similar effect as the stem VC benefited more from the NPK fertilization, while the root VC benefited more from the compost fertilization. In contrast, Han et al. (2016) found that in yellow poplar seedlings, both NPK and manure contribute similarly to stem and root growth. The observed differences could be partially due to the different composition of the fertilizers and application (Abubaker et al., 2020). However, as to the best of our knowledge, the impact of fertilization on the these two species as well as on the VC have not been studied and further research will be needed. In poplar, increasing the water availability when no fertilization is applied, indeed favored the stem CCn and CCl. These results also confirm a previous study which noted that poplar prioritizes above-ground development and more rapidly forms ground cover (Byambadorj et al., 2021a). The impact of compost fertilization on the *P. sibirica* VC - increased CCn and CCl in both the stem and the root – showed to be effective only when the water availability is increased, although in these conditions there is no consistently significant difference between the stem and root VC, unlike in conditions of control-irrigation. Thus, our initial hypothesis for the increase of the VC traits due to increase of the water regime, as well as its magnification of the fertilization effect, can be accepted in conditions when no fertilization was applied and with the compost treatment. However, when NPK-treatment was applied, the hypothesis is rejected as we observe increase in the root VC traits rather than the stem, and underperformance of the highest irrigation regime. The latter results were quite interesting - the highest irrigation of 8 L h^-1^ underperformed compared to the 4 L h^-1^ irrigation regime – as they indicate that the maximal capacity of the VC could be obtained at a lower irrigation level in both the stem and the root. In *U. pumila*, the VC traits did not differ between the control-irrigation and the highest irrigation of 8 L h^-1^, regardless of the fertilization regime. Furthermore, there was no clear consistency of a higher increase of both CCn and CCl in the stem rather than the root. These results could indicate that *U. pumila*, due to the lack of impact on the VC traits, is more adapted to the semi-arid regions. The inconsistent and limited impact on the management regimes on the VC traits, in particular the CCn, could be due to the fact that as the CCn depends on the tree species, age and vitality (Larson, 1994), the impact of the management treatments is not enough strong and/or consistent to significantly change the CCn. A more immediate impact is the change in the CCl which in the present study is only due to the compost – by increasing the root CCl and diminishing the stem CCl. However, more studies in the semi-arid areas would be needed to understand the VC range and traits.

### 4.2 Anatomical wood traits

The analysis of the measured anatomical wood traits showed that in *P. sibirica* the LWI is most impacted by the management regimes. Without additional irrigation and regardless of the fertilization treatment, the stem LWI benefited more than the root, and, both NPK and compost, were found to diminish the LWI. However, neither the EWXA nor the LWXA were significantly altered due to fertilization. These findings are in line with previous observations regarding the lack of impact of nitrogen fertilization on the stem vessel diameter in juvenile poplar (Luo et al., 2005) but in contrast with the positive correlation between potassium and the increase of vessel size in poplar stem (Fromm, 2010). Previous studies from our group have indicated that the stem biomass of poplar is not impacted by fertilization without irrigation (Byambadorj et al., 2021b). This is further confirmed by the present study, since when both NPK and compost treatment were used, the stem LWI benefited only when the water availability increased, thus allowing us to accept the initial hypothesis. However, when no fertilization was applied, all three irrigation regimes (2, 4, and 8 L h^-1^) contributed to a similar increase in stem LWI, indicating that water availability rather than fertilization has a bigger impact on the stem LWI. Since none of the anatomical wood traits (LWI, EWXA, LWXA) of the poplar root significantly differed, the present study is also in line with previous results that showed the root biomass in *P. sibirica* is not impacted by the fertilization regimes when no additional irrigation regime is applied (Nyam-Osor et al., 2021). Unlike in the stem, in *P. sibirica* root, the interplay of fertilization and irrigation impacted more the EWXA and LWXA rather than the LWI. Due to this different impact on the stem and root of *P. sibirica*, we could assume that when nutrients and water availability increase, the root prioritizes formations of larger xylem vessels (EWXA and LWXA) which would be able to transport the resources more efficiently in the stem where biomass increase, and wood production (LWI) is prioritized. It is well established that in temperate and (sub) tropical regions, earlywood vessels, formed in the spring, are wider than the latewood vessels form later in the growing season when less water is usually available (Baas et al., 1983; Pratt et al., 2015). We found that the same pattern is also present the *P. sibirica*, a diffuse-porous species, grown in the semi-arid climate, as consistently, regardless of the plant organ (stem or root) and the management combination (irrigation regime and fertilization treatment), the EWXA was higher than the LWXA. These results reflect the conditions since the start of the growing season (May) is characterized by increased water availability and temperature, the two main conditions that trigger wood formation, i.e., bigger vessels that maximize water transportation (Rahman et al., 2022). Although in arid conditions (high temperature, low water), the LWXA reduction along with reduced radial growth is a noted strategy for efficient water transportation (Islam et al., 2018), the vessel diameter is not necessarily the most suitable proxy for predicting some of the determinants of reduced plasticity and adaptability, i.e., drought-induced embolism (Lens et al., 2022). In *U. pumila*, the management regimes impacted only the length of the last wood increment. The higher length of the last wood increment in the stem vs the root in compost-treated *U. pumila*, allows us to accept the initial hypothesis only partially regarding the individual impact of fertilization in this species, as this was the only significant difference between all three anatomical wood traits (LWI, EWXA, and LWXA) of *U. pumila* stem and the root when analyzing the individual impact of fertilization. Previous results have shown that the lack of fertilization impact on *U. pumila* taproot, from where the microcore samples were obtained, as more biomass is allocated in roots with small and medium diameters rather than the taproot (Nyam-Osor et al., 2021).

The combined effect of fertilization and irrigation did not significantly impact any of the anatomical woody traits of the root of *U. pumila*, as no difference was observed between control - irrigation (0 L h^-1^) and the highest irrigation regime (8 L h^-1^). In the elm stem, the combined effect of fertilization and irrigation was most evident when no fertilizer or NPK were used. Interestingly, when no fertilization was used, we observed a proportional increase of the stem LWI as the water availability increased. However, in NPK-treated *U. pumila*, such a proportional increase was not the case as the 2 L h^-1^ irrigation contributed to a higher LWI than the more intensive irrigation regimes (4 and 8 L h^-1^). These findings support similar observations of the lack of benefit for *U. pumila* from more water regarding higher total biomass, root biomass and general survival (Byambadorj et al., 2021a). Regardless of the management regime, the length of the last wood increment was higher in the stem compared to the root, which partially confirms our hypothesis. In the stem, compost had the highest impact when combined with control irrigation, while in the root when combined with the maximal irrigation. Neither the EWXA nor the LWXA in *U. pumila* were impacted by the combination of the management regimes. The EW and LW vessels traits (size, length, density) dependent on the species and the growth conditions. As previously mentioned, the species in the present study are different, as *P. sibirica* is a diffuse-porous species, and *U. pumila* is a ring-porous species. These anatomical differences are also associated with a different strategy regarding the wood production, since ring-porous species use resources from the previous year for the EW production, and the diffuse-porous species are less dependent on it, thus reflecting the current growth year conditions better (Barbadoux and Breda 2002; Fonti and Garcia-Gonzalez, 2004). Thus, the lack of treatment impact on the EWXA and LWXA of *U. pumila*, and no difference between the stem and the root, could be an indicator of higher adaptability and plasticity, via its capacity to conserve resources that would support the start wood production (EWXA) and better use of the available resources during the growth period (LWXA).

## 5 Conclusions and future perspectives

In conclusion, our results indicate that, regardless of the species, the VC traits are less impacted by the management regimes since the increase in the watering regimes did not contribute to a significant VC trait increase, and the use of fertilizers did not contribute to a VC trait decrease. On the contrary, for both species, the length of the last wood increment was shown to be most sensitive to the changes in nutrients and water availability. As hypothesized, in *P. sibirica*, the length of the last wood increment benefited from the increased water availability to a higher extent, and primarily in the roots. This accurately reflects previous observations about the tendency of woody plants to allocate more resources in stem biomass. Furthermore, the reduced impact of the management regimes on both the VC and wood traits of *U. pumila* confirm the higher suitability of this species for semi-arid areas. The considered VC and wood traits of *U. pumila* did not benefit as much from the increased water availability, and the fertilization treatments had minor to no impact. To the best of our knowledge this is the first study that focuses in-depth on the vascular cambium and the anatomical wood traits of *Populus sibirica* and *Ulmus pumila*. Considering the very limited knowledge on wood anatomy in semiarid areas, future studies which include microcore analysis could indeed serve to understand our findings better and provide insight on the adaptability and plasticity of woody species.

## Data availability statement

The original contributions presented in the study are included in the article/**Supplementary Material.** Further inquiries can be directed to the corresponding author.

## Author contributions

AD: Data curation, Formal analysis, Methodology, Visualization, Writing – original draft, Writing – review & editing. AB: Data curation, Formal analysis, Methodology, Validation, Writing – review & editing. ET: Investigation, Methodology, Writing – review & editing. SB: Investigation, Methodology, Writing – review & editing. BN-O: Conceptualization, Funding acquisition, Investigation, Methodology, Writing – review & editing. GS: Funding acquisition, Supervision, Writing – review & editing. MM: Formal analysis, Methodology, Writing – review & editing. DC: Conceptualization, Investigation, Methodology, Validation, Writing – review & editing. AM: Conceptualization, Investigation, Methodology, Supervision, Validation, Writing – review & editing.

## References

[B1] Abubaker J. Ibrahim N. Alkanami M. Alaswd A. El-Zeadani H. (2020). Response of winter wheat to the application rate of raw and digested sheep manure alone and supplemented with urea in Libyan desert soil. Sci. Afr. 8, e00332. doi: 10.1016/j.sciaf.2020.e00332

[B2] Angerer J. Han G. Fujisaki I. Havstad K. (2008). Climate change and ecosystems of Asia with emphasis on Inner Mongolia and Mongolia. Rangelands 30, 46–51. doi: 10.2111/1551-501X(2008)30[46:CCAEOA]2.0.CO;2

[B3] Arnič D. Gričar J. Jevšenak J. Božič G. Von Arx G. Prislan P. (2021). Different wood anatomical and growth responses in European beech (*Fagus sylvatica* L.) at three forest sites in Slovenia. Front. Plant Sci. 12. doi: 10.3389/fpls.2021.669229 PMC8349990 34381473

[B4] Avirmed T. Byambadorj S.-O. Chiatante D. Sharavdorj K. Ganbat B. Sukhbaatar G.. (2023). Afforestation of semi-arid regions of Mongolia: carbon sequestration in trees and increase of soil organic carbon. Plant Biosyst. - Int. J. Deal. Asp. Plant Biol. 157, 779–791. doi: 10.1080/11263504.2023.2200781

[B5] Baas P. Werker E. Fahn A. (1983). Some ecological trends in vessel characters. IAWA J. 4, 141–159. doi: 10.1163/22941932-90000407

[B6] Balzano A. Battipaglia G. De Micco V. (2019). Wood-trait analysis to understand climatic factors triggering intra-annual density-fluctuations in co-occurring Mediterranean trees. IAWA J. 40, 241–258. doi: 10.1163/22941932-40190220

[B7] Balzano A. Čufar K. Battipaglia G. Merela M. Prislan P. Aronne G.. (2018). Xylogenesis reveals the genesis and ecological signal of IADFs in *Pinus pinea* L. and *Arbutus unedo* L. Ann. Bot. 121, 1231–1242. doi: 10.1093/aob/mcy008 29415209 PMC5946860

[B9] Batkhishig O. (2016). Soil classification of Mongolia. Mongolian J. Soil Sco. 1, 18–31.

[B10] Battipaglia G. Kabala J. P. Pacheco-Solana A. Niccoli F. Bräuning A. Campelo F.. (2023). Intra-annual density fluctuations in tree rings are proxies of air temperature across Europe. Sci. Rep. 13, 12294. doi: 10.1038/s41598-023-39610-8 37516810 PMC10387074

[B11] Byambadorj S.-O. Chiatante D. Akhmadi K. Lunten J. Ochirbat B. Park B. B.. (2021a). The effect of different watering regimes and fertilizer addition on the growth of tree species used to afforest the semi-arid steppe of Mongolia. Plant Biosyst. - Int. J. Deal. Asp. Plant Biol. 155, 747–758. doi: 10.1080/11263504.2020.1779845

[B12] Byambadorj S.-O. Nyam-Osor B. Park B. B. Avirmed T. Scippa G. S. Chiatante D.. (2021b). Afforestation of Mongolian steppe: patterns of biomass partitioning in *Populus sibirica* and *Ulmus pumila* trees in response to management supporting measures. Plant Biosyst. - Int. J. Deal. Asp. Plant Biol. 156, 969–981. doi: 10.1080/11263504.2021.1985002

[B13] Byambadorj S.-O. Park B. B. Hernandez J. O. Dulamsuren N. Sainbuyan Z. Altantugs O.. (2021c). Optimal irrigation regime for woody species potentially suitable for effective and sustainable afforestation in the desert region of Mongolia. Land 10, 212. doi: 10.3390/land10020212

[B14] Byambadorj S.-O. Park B. B. Hernandez J. O. Tsedensodnom E. Byambasuren O. Montagnoli A.. (2021d). Effects of irrigation and fertilization on the morphophysiological traits of *Populus sibirica* Hort. Ex Tausch and *Ulmus pumila* L. in the semiarid steppe region of Mongolia. Plants 10, 1–14. doi: 10.20944/preprints202110.0154.v1 PMC8620301 34834771

[B15] Chiatante D. Montagnoli A. Trupiano D. Sferra G. Bryant J. Rost T. L.. (2021). Meristematic connectome: a cellular coordinator of plant responses to environmental signals? Cells 10, 1–14. doi: 10.3390/cells10102544 PMC8533771 34685524

[B16] Čufar K. Cherubini M. Gričar J. Prislan P. Spina S. Romagnoli M. (2011). Xylem and phloem formation in chestnut ( growing season. Castanea sativa Dendrochronologia 29, 127–134. doi: 10.1016/j.dendro.2011.01.006

[B17] D’Andrea E. Rezaie N. Prislan P. Gričar J. Collalti A. Muhr J.. (2020). Frost and drought: Effects of extreme weather events on stem carbon dynamics in a Mediterranean beech forest. Plant Cell Environ. 43, 2365–2379. doi: 10.1111/pce.13858 32705694

[B18] De Luis M. Gričar J. Čufar K. Raventós J. (2007). Seasonal dynamics of wood formation in *Pinus halepensis* from dry and semi-arid ecosystems in Spain. IAWA J. 28, 389–404. doi: 10.1163/22941932-90001651

[B19] De Micco V. Balzano A. Čufar K. Aronne G. Gričar J. Merela M.. (2016). Timing of false ring formation in *Pinus halepensis* and *Arbutus unedo* in southern Italy: outlook from an analysis of xylogenesis and tree-ring chronologies. Front. Plant Sci. 7. doi: 10.3389/fpls.2016.00705 PMC4877369 27252721

[B20] Díaz S. Kattge J. Cornelissen J. H. C. Wright I. J. Lavorel S. Dray S.. (2016). The global spectrum of plant form and function. Nature 529, 167–171. doi: 10.1038/nature16489 26700811

[B21] Dimitrova A. Balzano A. Čufar K. Scippa G. S. Merela M. Montagnoli A.. (2023). Anatomy of xylem and phloem in stems and roots of *Populus sibirica* and *Ulmus pumila* from semi-arid steppe in Mongolia. Les/Wood 72, 37–48. doi: 10.26614/les-wood.2023.v72n02a02

[B22] Dov Y. B. Forti M. Pauker R. Aronson J. A. Pasternak D. (2001). “Introduction and selection of drought and salt tolerant plants for afforestation and landscaping in arid lands,” in Combating desertification with plants. Eds. Pasternak D. Schlissel A. (Springer US, Boston, MA), 121–148. doi: 10.1007/978-1-4615-1327-8_12

[B23] Fonti P. Garcia-Gonzalez I. (2004). Suitability of chestnut earlywood vessels chronologies from ecological studies. New Phytol. 163, 77–86. doi: 10.1111/j.1469-8137.2004.01089.x 33873786

[B24] From F. Strengbom J. Nordin A. (2015). Residual long-term effects of forest fertilization on tree growth and nitrogen turnover in boreal forest. Forest 6, 1145–1156. doi: 10.3390/f6041145

[B25] Fromm J. (2010). Wood formation of trees in relation to potassium and calcium nutrition. Tree Physiol. 30, 1140–1147. doi: 10.1093/treephys/tpq024 20439254

[B26] Galvin K. A. Reid R. S. Behnke R. H. Jr. Hobbs N. T. (2008). Fragmentation in Semi-Arid and Arid Landscapes: consequences for human and natural systems (Dordrecht: Springer). doi: 10.1007/978-1-4020-4906-4

[B27] Giagli K. Gričar J. Vavrčík H. Menšík L. Gryc V. (2016). The effects of drought on wood formation in *Fagus sylvatica* during two contrasting years. IAWA J. 37, 332–348. doi: 10.1163/22941932-20160137

[B28] Hag Husein H. Lucke B. Bäumler R. Sahwan W. (2021). A contribution to soil fertility assessment for arid and semi-arid lands. Soil Syst. 5, 42. doi: 10.3390/soilsystems5030042

[B29] Han S. H. An J. Y. Hwang J. Kim S. B. Park B. B. (2016). The effects of organic manure and chemical fertilizer on the growth and nutrient concentrations of yellow poplar (*Liriodendron tulipifera* Lin.) in a nursery system. For. Sci. Technol. 12, 137–143. doi: 10.1080/21580103.2015.1135827

[B30] Hilker T. Natsagdorj E. Waring R. H. Lyapustin A. Wang Y. (2014). Satellite observed widespread decline in Mongolian grasslands largely due to overgrazing. Glob. Change Biol. 20, 418–428. doi: 10.1111/gcb.12365 23966315

[B31] IAWA (1964). Multilingual glossary of terms used in wood anatomy (Winterhur, Switzerland: Verlaganstadt Buchdruckerei Konkordia).

[B32] InsideWood (2023) 2004-onwards. Available online at: http://insidewood.lib.ncsu.edu/search.

[B33] Islam M. Rahman M. Bräuning A. (2018). Xylem anatomical responses of diffuse porous *Chukrasia tabularis* to climate in a South Asian moist tropical forest. For. Ecol. Manage. 412, 9–20. doi: 10.1016/j.foreco.2018.01.035

[B34] Karlova R. Boer D. Hayes S. Testerink C. (2021). Root plasticity under abiotic stress. Plant Physiol. 187, 1057–1070. doi: 10.1093/plphys/kiab392 34734279 PMC8566202

[B35] Khaulenbek A. Kang H. (2017). “Collaboration project to combat desertification in Mongolia,” in PPP on the occasion of the International Conference on Environment and Technology, 27.10.2017, Ulaanbaatar, Mongolia.

[B36] Landsberg J. J. Sands P. J. (2011). Physiological ecology of forest production: principles, processes and models. 1st ed (Amsterdam Boston: Academic Press/Elsevier).

[B37] Larson P. R. (1994). The vascular cambium (Berlin, Heidelberg: Springer Berlin Heidelberg). doi: 10.1007/978-3-642-78466-8

[B38] Lavrenko E. M. Karamysheva Z. V. Nikulina R. I. (1991). The steppes of eurasia (Stepi evrazii) (Leningrad: Nauka).

[B39] Lee D. Ahn G. (2016). A way forward to sustainable international forestry cooperation: a case study of the ‘greenbelt plantation project in Mongolia. J. Rural Dev./Nongchon-Gyeongje 39, 143–168. doi: 10.22004/AG.ECON.251932

[B40] Lens F. Gleason S. M. Bortolami G. Brodersen C. Delzon S. Jansen S. (2022). Functional xylem characteristics associated with drought-induced embolism in angiosperms. New Phyto 236, 2019–2036. doi: 10.1111/nph.18447 36039697

[B43] Liu Y. Y. Evans J. P. McCabe M. F. De Jeu R. A. M. Van Dijk A. I. J. M. Dolman A. J.. (2013). Changing climate and overgrazing are decimating Mongolian steppes. PloS One 8, e57599. doi: 10.1371/journal.pone.0057599 23451249 PMC3581472

[B41] Liu H. Wang H. Li N. Shao J. Zhou X. Van Groenigen K. J.. (2022a). Phenological mismatches between above- and belowground plant responses to climate warming. Nat. Clim. Change 12, 97–102. doi: 10.1038/s41558-021-01244-x

[B42] Liu H. Xu C. Allen C. D. Hartmann H. Wei X. Yakir D.. (2022b). Nature-based framework for sustainable afforestation in global drylands under changing climate. Glob. Change Biol. 28, 2202–2220. doi: 10.1111/gcb.16059 34953175

[B44] Luo Z. B. Langenfeld-Heyser R. Calfapietra C. Polle A. (2005). Influence of free air CO_2_ enrichment (EUROFACE) and nitrogen fertilisation on the anatomy of juvenile wood of three poplar species after coppicing. Trees 19, 109–118. doi: 10.1007/s00468-004-0369-0

[B45] Miranda J. C. Calderaro C. Cocozza C. Lasserre B. Tognetti R. Von Arx G. (2022). Wood anatomical responses of European beech to elevation, land use change, and climate variability in the Central Apennines, Italy. Front. Plant Sci. 13. doi: 10.3389/fpls.2022.855741 PMC8983936 35401623

[B46] Montagnoli A. Dumroese R. K. Terzaghi M. Onelli E. Scippa G. S. Chiatante D. (2019). Seasonality of fine root dynamics and activity of root and shoot vascular cambium in a *Quercus ilex* L. forest (Italy). For. Ecol. Manage. 431, 26–34. doi: 10.1016/j.foreco.2018.06.044

[B47] Montagnoli A. Lasserre B. Terzaghi M. Byambadorj S.-O. Nyam-Osor B. Scippa G. S.. (2022). Fertilization reduces root architecture plasticity in *Ulmus pumila* used for afforesting Mongolian semi-arid steppe. Front. Plant Sci. 13. doi: 10.3389/fpls.2022.878299 PMC9359110 35958214

[B48] Monteoliva S. E. Villegas M. S. Achinelli F. G. (2015). Short-term and long-effects of weed control and fertilization on growth and wood anatomy of a *Populus deltoides* clone. For. Syst. 24, e005. doi: 10.5424/fs/2015241-05077

[B49] Nyam-Osor B. Byambadorj S.-O. Park B. B. Terzaghi M. Scippa G. S. Stanturf J. A.. (2021). Root biomass distribution of *Populus sibirica* and *Ulmus pumila* afforestation stands is affected by watering regimes and fertilization in the Mongolian semi-arid steppe. Front. Plant Sci. 12. doi: 10.3389/fpls.2021.638828 PMC8102691 33968099

[B50] Pasho E. Julio Camarero J. Vicente-Serrano S. M. (2012). Climatic impacts and drought control of radial growth and seasonal wood formation in *Pinus halepensis* . Trees 26, 1875–1886. doi: 10.1007/s00468-012-0756-x

[B51] Perez-de-Lis G. Olano J. M. Rozas V. Rossi S. Vázquez-Ruiz R. A. García-González I. (2017). Environmental conditions and vascular cambium regulate carbon allocation to xylem growth in deciduous oaks. Funct. Ecol. 31, 592–603. doi: 10.1111/1365-2435.12789

[B52] Plavcová L. Hacke U. G. (2012). Phenotypic and developmental plasticity of xylem in hybrid poplar saplings subjected to experimental drought, nitrogen fertilization, and shading. J. Exp. Bot. 63, 6481–6491. doi: 10.1093/jxb/ers303 23095999 PMC3504499

[B53] Pratt R. B. Percolla M. I. Jacobsen A. L. (2015). “Integrative xylem analysis of chaparral shrubs,” in Functional and ecological xylem anatomy. Ed. Hacke U. (Springer International Publishing, Cham), 189–207. doi: 10.1007/978-3-319-15783-2_7

[B54] Prislan P. Čufar K. De Luis M. Gričar J. (2018). Precipitation is not limiting for xylem formation dynamics and vessel development in European beech from two temperate forest sites. Tree Physiol. 38, 186–197. doi: 10.1093/treephys/tpx167 29325135

[B55] Prislan P. Gričar J. Čufar K. De Luis M. Merela M. Rossi S. (2019). Growing season and radial growth predicted for *Fagus sylvatica* under climate change. Clim. Change 153, 181–197. doi: 10.1007/s10584-019-02374-0

[B56] Prislan P. Gričar J. De Luis M. Smith K. T. Čufar K. (2013). Phenological variation in xylem and phloem formation in *Fagus sylvatica* from two contrasting sites. Agric. For. Meteorol. 180, 142–151. doi: 10.1016/j.agrformet.2013.06.001

[B57] Querejeta J. I. Roldán A. Albaladejo J. Castillo V. (2001). Soil water availability improved by site preparation in a *Pinus halepensis* afforestation under semiarid climate. For. Ecol. Manage. 149, 115–128. doi: 10.1016/S0378-1127(00)00549-1

[B59] Rahman M. H. Begum S. Nugroho W. D. Nakaba S. Funada R. (2022). The effects of watering on cambial activity in the stems of evergreen hardwood (*Samanea saman)* during the pre-monsoon season in subtropical Bangladesh. J. Wood Sci. 68, 47. doi: 10.1186/s10086-022-02053-2

[B58] R Core Team (2021). R: A language and environment for statistical computing (Vienna, Austria: R Foundation for Statistical Computing).

[B60] Reisman-Berman O. Keasar T. Tel-Zur N. (2019). Native and non-native species for dryland afforestation: bridging ecosystem integrity and livelihood support. Ann. For. Sci. 76, 114. doi: 10.1007/s13595-019-0903-2

[B61] Ren P. Rossi S. Gricar J. Liang E. Cufar K. (2015). Is precipitation a trigger for the onset of xylogenesis in *Juniperus przewalskii* on the north-eastern Tibetan Plateau? Ann. Bot. 115, 629–639. doi: 10.1093/aob/mcu259 25725006 PMC4343293

[B62] Rosell J. A. Olson M. E. Anfodillo T. (2017). Scaling of xylem vessel diameter with plant size: causes, predictions, and outstanding questions. Curr. For. Rep. 3, 46–59. doi: 10.1007/s40725-017-0049-0

[B63] Rossi S. Anfodillo T. Menardi R. (2006). Trephor: a new tool for sampling microcores from tree stems. IAWA J. 27, 89–97. doi: 10.1163/22941932-90000139

[B64] Schneider C. A. Rasband W. S. Eliceiri K. W. (2012). NIH Image to ImageJ: 25 years of image analysis. Nat. Methods 9, 671–675. doi: 10.1038/nmeth.2089 22930834 PMC5554542

[B65] Singh P. K. Chudasama H. (2021). Pathways for climate change adaptations in arid and semi-arid regions. J. Clean. Prod. 284, 124744. doi: 10.1016/j.jclepro.2020.124744

[B66] Smolander A. Henttonen H. M. Nöjd P. Soronen P. Mäkinen H. (2022). Long-term response of soil and stem wood properties to repeated nitrogen fertilization in a N-limited Scots pine stand. Eur. J. For. Res. 141, 421–431. doi: 10.1007/s10342-022-01448-6

[B67] Ulziykhutag N. (1989). Overview of the flora of Mongolia (Ulaanbaatar: State Publishing).

[B68] Van Der Werf G. W. Sass-Klaassen U. G. W. Mohren G. M. J. (2007). The impact of the 2003 summer drought on the intra-annual growth pattern of beech (*Fagus sylvatica* L.) and oak (*Quercus robur* L.) on a dry site in the Netherlands. Dendrochronologia 25, 103–112. doi: 10.1016/j.dendro.2007.03.004

[B70] Wheeler E. A. (2011). InsideWood – a web resource for hardwood identification. IAWA J. 32, 199–211. doi: 10.1163/22941932-90000051

[B71] Wheeler E. A. Gasson P. E. Baas P. (2020). Using the InsideWood web site: potentials and pitfalls. IAWA J. 41, 412–462. doi: 10.1163/22941932-bja10032

[B69] Yosef G. Walko R. Avisar R. Tatarinov F. Rotenberg E. Yakir D. (2018). Large-scale semi-arid afforestation can enhance precipitation and carbon sequestration potential. Sci. Rep. 8, 996. doi: 10.1038/s41598-018-19265-6 29343760 PMC5772497

